# *In Silico* Study of Mercury Resistance Genes Extracted from *Pseudomonas* spp. Involved in Bioremediation: Understanding the Promoter Regions and Regulatory Elements

**DOI:** 10.1155/2022/6185615

**Published:** 2022-08-12

**Authors:** Duguma Dibbisa, Gobena Wagari

**Affiliations:** ^1^School of Biological Sciences and Biotechnology, Haramaya University, Dire Dawa, Ethiopia; ^2^Department of Animal Science, Oda Bultum University, Chiro, Ethiopia

## Abstract

Microbial genes and their product were diverse and beneficial for heavy metal bioremediation from the contaminated sites. Screening of genes and gene products plays a significant role in the detoxification of pollutants. Understanding of the promoter region and its regulatory elements is a vital implication of microbial genes. To the best of our knowledge, there is no in silico study reported so far on *mer* gene families used for heavy metal bioremediation. The motif distribution was observed densely upstream of the TSSs (transcription start sites) between +1 and -350 bp and sparsely distributed above -350 bp, according to the current study. MEME identified the best common candidate motifs of TFs (transcription factors) binding with the lowest e value (7.2*e*-033) and is the most statistically significant candidate motif. The EXPREG output of the 11 TFs with varying degrees of function such as activation, repression, transcription, and dual purposes was thoroughly examined. Data revealed that transcriptional gene regulation in terms of activation and repression was observed at 36.4% and 54.56%, respectively. This shows that most TFs are involved in transcription gene repression rather than activation. Likewise, EXPREG output revealed that transcriptional conformational modes, such as monomers, dimers, tetramers, and other factors, were also analyzed. The data indicated that most of the transcriptional conformation mode was dual, which accounts for 96%. CpG island analysis using online and offline tools revealed that the gene body had fewer CpG islands compared to the promoter regions. Understanding the common candidate motifs, transcriptional factors, and regulatory elements of the *mer* operon gene cluster using a machine learning approach could help us better understand gene expression patterns in heavy metal bioremediation.

## 1. Introduction

Worldwide, human populations are increasing at alarming rates. It has been estimated that this population will reach nine billion by the year 2050 [1, 2]. Population growth contributes to the degradation of natural resources. Thus, environmental protection is imperative for a functioning and balanced ecosystem. Several environmental pollutants cause multifaceted degradation and affect ecosystem components, particularly soil, water, and the entire biodiversity. Heavy metals chemically refer to a class of specific subdivisions of elements marked with metallic properties. It is the most significant atmospheric contaminant discharged from natural and anthropogenic activities. Metals are everywhere but in different concentrations. Exceeding the required concentration will result in contamination [3]. The density at 5 gcm^−3^ and the concentrations of heavy metals present in the environment are highly toxic to biodiversity [4].

The availability or entry of heavy metals into the ecosystem comes from various sources, either naturally or human-induced activities. The natural sources of heavy metal contamination include geological weathering, volcanic eruptions, industrial effluents, and chemicals widely used in the agricultural sectors, namely, pesticides, herbicides, and insecticides are sources of anthropogenic activities [5]. Our natural environment is also contaminated by heavy corrosion, metal ions, heavy metal leaching, and household wastes released into the soil and groundwater. Gold mining and other metal industries are the main causes of soil contamination from mercury. Mercury is a unique important heavy metal extensively used in the developing and industrialized world for income. Nonetheless, the trend in developing countries is significantly lower; for example, in Ethiopia, it is a widespread practice in some areas [6]. Heavy metal like mercury is essential for living organisms in certain concentrations; however, its excessive concentrations are significantly carcinogenic and toxic. The toxicity of these heavy metals can cause severe illness in humans and animals [3].

The removal of heavy metals from the environment has become an extremely pertinent issue in the current scenario. The uses of different methods to remove or reduce the harmful effects of heavy metal contamination are physical evacuation, chemical cleaning, and stabilization of metals at the site, as well as the use of biological entities as bioremediation [7]. Using microbial biomass as a platform for heavy metal ion removal is an alternative method of bioremediation. It is a biological phenomenon in which microbes use genes and gene products to take up and accumulate metal ions in the intracellular space for use in cellular processes [8]. Heavy metal ions can be absorbed and accumulated by microorganisms in their intracellular space and used for a variety of purposes. Therefore, numerous studies focus on the cost-effective and environmentally friendly applications of bioremediation in heavy metal removal. Transcription factors (TFs) that recognize specific DNA sequences near promoter regions and transcription factor binding sites associated with genes that play key roles in the structure and function of genes and the region of promoter of genes in mercury bioremediation have not yet been studied. Therefore, the objective of this study was to identify the promoter region, transcriptional factors with corresponding binding sites, and CpG islands involved in the regulation of expression, to provide baseline information for working mercuric bioremediation for environmental applications.

## 2. Materials and Methods

### 2.1. Determination of TSS and Promoter Regions

The *Pseudomonas* spp. gene sequences responsible for mercuric bioremediation were retrieved from the NCBI genome browser that is available at https://www.ncbi.nlm.nih.gov/gene in March 2022 as in Table 1. For the current study, about ten protein-coding sequences were extracted after checking the search results in the sequence database. To analyze the specific gene further, the presence of the starting coding sequences was predicted whether they were found on positive or negative strands. The region of the transcriptional start site (TSS) was determined by extending sequences from the genomic coordinate regions. The FASTA file format of query sequences was used for further analysis. The prepared 1 kb upstream sequences from the start codons were taken to Neural Network Promoter Prediction (NNPP version 2.2) (https://www.fruitfly.org/seq_tools/promoter.html) tools to obtain the potential TSS [9]. The NNPP version 2.2 toolset was used with a minimum standard predictive promoter score with a default cutoff value of 0.8 for prokaryotic cells and intended to eliminate zero counts by 80% from the query sequences before transformation [9]. Based on the output of NNPP, promoter prediction sequence regions for those containing more than one TSS, the highest prediction score was considered for trustable and accuracy cutoff values. The remaining TSS regions were just utilized for simple comparative analysis [10–12].

### 2.2. Determination of Common Motifs and TFs in *Pseudomonas spp.* Genes

The promoter sequence regions identified based on the established criteria were imported and studied using the MEME (5.4.1 version) via the web server hosted by the National Biomedical Computational Resource (https://meme-suite.org/meme/tools/meme) [13] to look for common candidate motifs that serve for the binding sites of transcriptional factors that regulate the expression of heavy metal accumulated genes. MEME suite searches for statistically significant candidate motifs in the sequence that was imported. The MEME suite predicted and discovered gene sequences with novel motifs (fixed-length repetitive patterns) were submitted to online tools. This technique determined the occurrence of common motifs that serve as binding sites for the transcription factors expected to regulate the expression levels of heavy metal bioaccumulation. MEME suite was used to perform motif prediction and discovery, motif alignment analysis, motif scanning, and motif comparison [14]. Before starting the search for typed sequences, the basic search parameters for the motif distribution menu were set, including the distribution of motif locations, zero options, or more occurrences per sequence, while keeping the number of motifs and the remaining motif width (6-50 bps) as the default. After the MEME searches were completed, the search result page was linked to the MEME output in HTML format. This stage is a fundamental initial point of view for the expected value (*e* value). The smaller the *e* value, the better the agreement [14]. At the bottom of the MEME HTML output, one or all candidate motifs can be forwarded for further analysis and the identical motifs can be further characterized by other web server programs. In these cases, the TOMTOM web server was used to search for sequences that matched the identified motif in its respective TFs. TOMTOM output includes LOGOSS representing the alignment of the candidate motif and TF with the *p* value and *q* value (a measure of the false discovery rate) of the match and links back to the parent transcription database for more detailed sequence match information [14, 15].

### 2.3. Search for CpG Islands for *Pseudomonas* spp. Encoding Genes

A 2 kb query sequence in FASTA format from the upstream of the start codon was prepared for all ten *Pseudomonas spp.* protein-coding gene sequences. The regulatory region, CpG islands representing regions of a sequence, was examined with two algorithms. The first algorithm was the offline tool CLC Genomic Workbench version 20.0.40, CLC Bio, Aarhus, Denmark) used to search the restriction enzyme sites *MspI*, with fragment sizes between 40 and 220 bp parameters. The second tool was the Takai and Jones algorithm with search criteria of GC contents of ≥55% and observed CpG/expected CpG ratio of ≥0.65% and a length of ≥500 bp [16]. The CpG island search tool available at the web link (http://dbcat.cgm.ntu.edu.tw/) was used for this purpose.

## 3. Results

### 3.1. Determination of Transcriptional Start Sites (TSSs)

Understanding a regulatory element is one of the most difficult challenges in the entire genome. Therefore, identification of the TSS is the key information for gene expression. Transcription start sites (TSSs) are the first nucleotides of DNA sequences where transcription has been started. On the other hand, it is where the RNA polymerase enzyme binds upstream of the start site. The online Neural Network Promoter Prediction (NNPP) version 2.20 databases were used to find the TSS for the gene extracted from *Pseudomonas* spp., which is widely used for mercury bioremediation. The promoter region located upstream of 1 kb of the TSS was characterized on the assumption that the functional gene elements of the promoter can be found within the region. The TSSs predicted values for each of the coding sequences of *mer* operon gene varieties in mercury bioremediation have been summarized and presented in Table 2. Accordingly, the *mer* operon gene variety has several TSS values ranging from 1 to 4. Interestingly, about six identified genes (*merA*, *merB*, *merD*, *merE*, *merF*, and *merP*) have the same TSS values, and *merC* has only one TSS value as can be seen in Table 2.

The TSSs were located at various distances from the start codon, having a maximum and minimum of 2921 and 409, respectively, as observed in Table 2.This variation of location of the start codon was enhancing or hindering transcriptional initiation and its gene regulation. The genes indicated by *merD*, *merG*, and *merR* were the highest values observed for positive-strand localization, respectively, while *merB* and *MerE* were the highest values that have been among the other TSS found on the negative strands. However, the majority of the TSS of *mer* operon genes was found on the negative strand, while few of them were on the positive strands. Understanding TSS applications such as gene function and its structure, predicting promoter regions and gene regulation has been apparent in the current gene prediction scenario (Table 2).

### 3.2. Determination of Common Motifs and TFs

The five candidate motifs were predicted and investigated by the MEME algorithm as shown in Table 3. Ten imported thousand-length gene sequences were analyzed to generate the five most promising candidate motifs. The predicted motifs and the proportion of promoters containing common motifs for the *mer* operon gene were evaluated. The data show that the most common motifs (motif_1) with the lowest *e* values have 100% binding sites. The predicted candidate motifs have the lowest (motif_5) and highest (motif_1) *e* values, 7.2*e*-033 and 7.3*e*-074, respectively. Therefore, the most likely candidate (motif 1) has the highest binding sites compared to the other candidate motifs. As presented in Table 3, the two common candidate motifs (motif_2 and motif_3) shared binding sites and had common motif width by variation in the *e* values.

**Figure 1:**
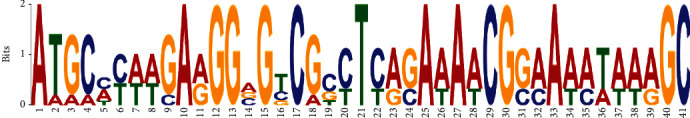
Sequence logos for mercuric bioremediation identified common motifs. The analysis was done by the MEME suite.

A candidate common motif with the lowest *e* value (7.2*e*-033) represents a statistically significant and functionally significant motif imported into TOMTOM version 5.4.1 for further analysis (https://meme-suite.org/meme/doc/tomtom-output-format.htmll), which is a publicly available database for transcription factor prediction that could be similar to known regulatory motifs [14, 15]. TOMTOM provides LOGOSS representing the alignment of the known motifs with the candidate transcription factors. The TOMTOM output from the database includes links to the parental TF database for more information such as activation, repression, and dual regulatory roles of the matched motifs (Table 4). Again, there was also other conformational information associated with the TF databases such as monomers, dimers, tetramers, and unidentified as well as other factors. The binding types associated with the databases were also predicted. The motif_5 had the lowest *e* values (7.2*e*-033) and statistically significant with 11 matched TFs from 84 collected databases with matched *e* value thresholds less than 10 or less as screened and observed from the TOMTOM database. The forward and reverse strands of the statistically significant strands are depicted in Figure 1.

**Figure 2:**
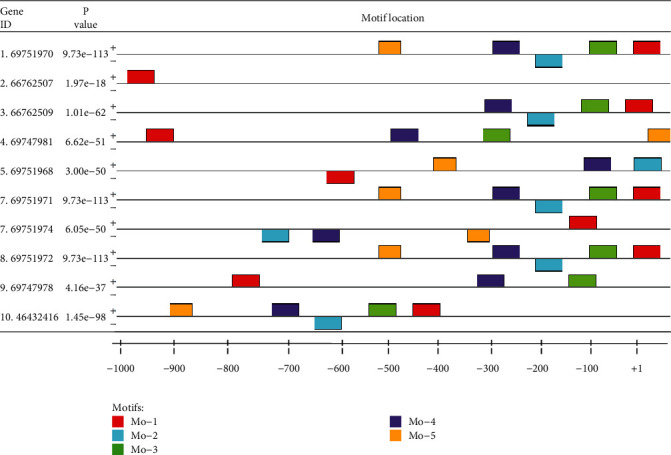
The relative locations of potential motifs in the promoter region relative to TSSs are illustrated in block diagrams. The nucleotide locations in the promoter region for *mer* genes encoding for mercury bioremediation are indicated at the bottom of the graph, ranging from +1 (start of TSSs) to upstream 1 kb (-1 kb) from MEME suite output.

For TSS, we checked the distribution from position +1 of the upstream to position -1 kb (Figure 2). Using the present analysis, the motif distributions (75% on the positive complement strands and 25% on the negative complement strands) are presented in Figure 2. They were distributed at each site according to the transcriptional start site. Additionally, the data indicates that the dense distribution of the common candidate motifs lies around the -350 to +1 bp region, while a few of them are distributed between -1 kb and -350 bp region; the relative location and spatial distribution of these motifs in the promoter regions were constructed by MEME and the created logos of common motifs, resulting in different characteristics of the column's motif orientations, with the height of the letter illustrating how frequently that nucleotide is expected to be observed in that particular position of the two strands (Figure 2). It has been suggested that the motifs found in many promoter regions could provide a significant amount of information [17].

Motifs have been revealed to be extremely beneficial in identifying genetic regulatory networks and interpreting specific gene activities. Regulatory motif discovery analysis has advanced significantly attributable to our current computational capabilities, and it remains at the forefront of genomic investigations of bacteria employed in environmental remediation. According to the current study, the identified candidate motif was widely dispersed between +1 and -350 bp, sparsely distributed between -350 and -800 bp, and less distributed above -800 bp as illustrated in Figure 2. The distribution was on both positive and negative strands, with transcription start sites as a reference. Only one candidate motif was found on the positive complementary strands in the gene identified by gene ID (66762507). Approximately 75% and 25% of the candidate motifs were located on the positive and negative strands, respectively. This indicates most of the candidate motifs were discovered on the positive strands. The variation of motif distribution is resulted from the difference in nucleotide sequences of the identified genes.

Identification of transcription factors is an essential regulator of gene expression, determining where and to what extent genes are expressed in molecular biology. As observed in Table 4, eleven transcriptional factors matching the candidate motif were discovered, each with different regulatory activities. From the commonly identified transcriptional factors, four (*PhhR* (90%), *VqsM* (7%), *CcpA* (1%), and *LrP* (1%)) have activation or regulatory roles with differences in degree. This study also revealed that only one *CtrA* (9.09%) and two, namely, *CRP* and *GlxR* (18.18%), TFs identified from *C. crescentus*, *Y. pestis*, and *C. glutamicumorganism* had dual and repression regulatory functions, respectively. Most of the TFs (*CodY*, *EspR*, *MatP*, and to some extent, *VqsM*, *Fur*, *Lrp*, and *CtrA*) have been found for activation of transcription for mercuric bioremediation and have not yet been described; therefore, additional wet lab-based research might be needed in the future.

Transcription factors regulate some sets of gene regulation, and conformational factors and flexibility of genes lead to an effective and selective assembly of coregulatory proteins to regulate the target genes. This indicates that the transitory interactions between TF and site-specific DNA sequences are common and important in biological functions. It could be hypothesized that these transcription factors activate gene regulatory roles in the bioremediation of environmental pollutants by mercury (II) reductase in the case of the *merA* gene, organomercury lyase (*merB*), mercury transporter gene (*merC*, *merE*, *merF*, and *merT*), transcription regulators (*merR*), and finally mercury-resistant genes (*merF*, *more*, and *merG*) as presented in Table 4.

Accordingly, the transcriptional factor confirmation mechanism of eleven *mer* genes employed in mercury bioremediation was studied. According to the current study, no regulatory role has been assigned to the complete set of candidate TFs, monomers, tetramers, or other conformational modes as indicated in Table 5. Approximately four of these (*PhhR*, *Fur*, *EspR*, *MatP*, and *Lrp*) discovered TF candidates have 100% and 96% dimer conformational roles in coregulating genes, respectively. The current investigation revealed that about 54.54% of the identified common candidates for TF conformational mechanisms' function were not identified in Table 5. The conformational flexibility of TF binding proteins maximizes gene regulatory efficiency.

### 3.3. Determination of CpG Islands

CpG islands are DNA methylation sites in promoter regions that are utilized as gene regulation tools by silencing a related gene during transcription. For this study, two algorithms, offline CLC Genome Workbench version 22.0.10 and online database search tools, were used. The two regions (promoter and gene body) were analyzed in FASTA format from the upstream of the start codon as well as the whole gene body sequences. Using online database searching tools, the analysis revealed that CpG islands exist in approximately 30% of the gene body and 70% of the promoter regions, respectively. The gene body sequences with gene IDs 46432416, 66762507, and 69751970 were among the genes with one CpG island each when compared to other genes. Similarly, gene IDs 46432416, 66762507, 69747978, 69747981, 69751970, 69751971, and 69751972 had one CpG island in the promoter regions. The data also revealed that 30% of the same gene bodies and promoter regions have common CpG islands while 30% have no CpG islands as depicted in Table 6.

Further investigations were done offline using CLC Genome Workbench version 22.0.10 to analyze the CpG islands. The restriction enzyme *MspI* was used in the second alternative, which revealed the presence of CpG islands in both promoter regions and gene bodies. As it was revealed in Table 7, the restriction enzyme *MspI* was used to cut fragments between 40 and 220 bp in the promoter region rather than the gene body. In general, the nucleotide cutting position of the promoter region was higher than the gene body. This indicated that poorer CpG islands were observed in the gene body than in the promoter regions.

## 4. Discussions

Bacterial genomes contain a wide range of genes, each with its function, composition, structure, replication, and transcription, which are used in molecular biology research [18]. Identifying the TSSs from the upstream of the gene as well as identifying the promoter region can play a significant role in understanding gene regulation mechanisms in microbial cells [10]. Ten common gene sequences used in mercuric bioremediation were retrieved from NCBI database in March 2022 for the current study. The results showed that the genes encoding mercury bioremediation were predicted and different in the TSS [11, 12]. Current studies show that the promoter region of all sequences had multiple TSS values, showing a similar investigation of genome-wide identification of TSS promoter and TF binding sites in *E. coli* [19].

The present study revealed that the dense distribution of TSSs values in mercuric bioremediation was found between +1 bp and -400 bp, as observed in Figure 2. Promoter regions were found to share the same patterns of motifs that function as binding sites for transcriptional factors (TF) to facilitate the gene regulation mechanism. If transcription is correctly initiated, the regulatory elements present upstream of the transcribed region are eventually required to determine gene regulation. In the current study, about 11 transcriptional factors that facilitate gene regulation in mercuric bioremediation were investigated and presented very well. The motif patterns in the promoter region, which operates the binding sites of transcription factors, could believe to enhance gene regulation [20]. *PhhR*, *VqsM*, *CcpA*, and *Lrp* were discovered to be involved in activation gene regulation role among the TFs identified using Uniprot database. According to numerous studies, the transcription analysis of the *PhhR* TF was important for controlling four putative transcriptional units such as *phhA*, *hpd*, *hmgA*, and *dhcA.* The current finding is in line with the previous findings of the transcriptional activation of the *PhhR* gene in *Pseudomonas aeruginosa* that is responsible for the transcriptional activation of genes for phenylalanine degradation [21].

From the analyzed results, transcriptional factors such as *CcpA*, *GlxR*, and *CRP* were widely used for transcriptional repression. The current findings were consistent with the catabolic repression mediated by *CcpA* in *B. subtilis* reported by Moreno and his colleagues [22], the negative regulation of *sycO*-*ypkA*, the *ypoJ* operon in *E. coli* by cyclic AMP [23], and the *GlxR* involved in the repression of *aceB*, which codes for malate synthase [24]. In the presence of a cAMP binding motif, *GlxR* TF shares common functions with the *CRP* in *E. coli*.

The *MspI* restriction enzyme was used to search for CpG islands in both the promoter and gene body regions were presented in Tables 7 and 8. The promoter region sequences, Prom_69751970, Prom_46432416, and Prom_66762507 and Prom_69751972, had the same *MspI* cleavage sites and fragment length in the current analysis. However, the locations of the TSS of each promoter sequences were different. The highest and lowest cutting sites of *MspI* were found in Prom_69751968 and Prom_66762509, respectively. In the gene body region, the highest and lowest *MspI* cutting sites were represented by ORF_69751970 and ORF_66762509, ORF_69751972, ORF_46432416, and ORF_69751971, respectively. The results of the *MspI* restriction enzyme digestion revealed that the promoter region had more CpG islands than its counterpart as seen in Tables 7 and 8. This result indicates that the promoter region of the *Mer* operon genes has rich CpG islands that play a crucial role in gene regulation applications while compared to the gene bodies as indicated above. Hande and his colleagues reported similar finding in the *Mycobacterium colombiense* CECT 3035 [25]. The current finding agreed with the finding of gene expression in the promoter-associated CpG islands in the human methylome [26].

The *mer* genome consists of ten essential *mer* gene clusters that play an imperative function in mercuric bioremediation. The mainstream of the *mer* gene sequences found in bacterial strains belongs to *gammaproteobacterial*, followed by *alphaproteobacterial*. Those gene groups were also discovered in beta *proteobacteria*, *firmicutes*, and *actinobacteria* to varying degrees. Each group of *mer* genomes performs a specific function. One of the major applications of *merA* was in reducing mercury from Hg^2+^ to Hg^0^, a process widely used in bioremediation, while *merB*, *merC*, and *merT* were important for organomercurial lyase and transporters, respectively. On the other hand, *merB* and *merE* were broad-spectrum *mer* operons found in both gram-positive and gram-negative bacteria used in mercuric bioremediation. *merD* and *merR* were among the *mer* gene clusters used in transcriptional regulation and coregulation of mercuric resistance in bioremediation, respectively, as depicted in Table 8. The *mer* genomes and their cluster genes, in general, have played a crucial role in the current scenario of environmental contamination control mechanisms. This study agreed with the study conducted on biogeochemistry and bioremediation of mercury by bacteria [27].

## 5. Conclusions

The current investigation and characterization of promoter regions of the *mer* genome and its gene clusters encoding mercuric heavy metal resistance as a means of mercuric bioremediations are particularly important for understating the regulatory elements and control of its expression. The current finding revealed that eleven transcriptional factors and their conformational modes identified in the promoter region of the *mer* operon gene clusters could play a major application in heavy metal bioremediation such as mercury. By contributing to improving environmental concerns caused by global climate change, the current study contributes to improving the environment. However, additional experimental studies will be required to confirm the role of the identified TFs and their shared binding locations in the regulation of the *mer* gene encoding for heavy metal bioremediation by using advanced bioinformatics tools to improve the effectiveness of the *mer* gene clusters.

## Figures and Tables

**Table 1:** Mercury bioremediation genes and their general function and genome coordinates.

SN	Gene ID	Gene symbol	Genome coordinate	Gene function
1.	69751970	*merA*	c33607-31961	Hg^2+^ reductase applications
2.	66762507	*merB*	c3805546-3806184	Organomercurial lyase
3.	66762509	*merC*	c3808349-3807915	Organomercurial transporter
4.	69747981	*merD*	188629-188994	Mercury resistance coregulator
5.	69751968	*merE*	c31582-31346	Broad-range mercury transporter
6.	69751971	*merF*	c33849-33604	Mercury resistance protein
7.	69751974	*merR*	34565-34999	Hg^2+^ responsive transcriptional regulator
8.	69751972	*merP*	c34127-33852	Mercury resistance system periplasmic binding protein
9.	69747978	*merT*	186216-186566	Mercuric ion transporter
10.	46432416	*merG*	5771173-5771826	Phenyl mercury resistance protein

Genes extracted from NCBI.

**Table 2:** TSS number, its promoter predictive score values, and distance from 5′UTR region of the corresponding gene.

SN	Gene ID	Gene symbol	No. of predictive promoter	No. of TSS identified	The predictive score value cut off at 0.80	5′UTR region size (bp)	Orientation of complementary strands
1.	69751970	*merA*	2	2	0.97, 0.91	-929	-ve
2.	66762507	*merB*	2	2	0.85, 0.82	-1951	-ve
3.	66762509	*merC*	1	1	0.85	-686	-ve
4.	69747981	*merD*	2	2	0.93, 0.89	2921	+ve
5.	69751968	*merE*	2	2	0.86, 0.94	-1361	-ve
6.	69751971	*merF*	2	2	0.97, 0.91	-687	-ve
7.	69751974	*merR*	4	4	0.97, 0.89, 0.89, 0.86	865	+ve
8.	69751972	*merP*	2	2	0.97, 0.91	-409	-ve
9.	69747978	*merT*	3	3	0.92, 0.93, 0.89	663	+ve
10.	46432416	*merG*	3	3	0.89, 0.94, 0.85	2217	+ve

NNPP tool prediction results are considered reliable at 0.8 cutoff values for the prokaryotic organism [9].

**Table 3:** List of predicted motifs and the number and proportion of promoter-containing motifs.

SN	Predicted and discovered candidate motifs	No. of the promoter for each of the motifs in %	*e* value^a^	Motif widths	No. of the binding sites
1.	Motif_1	10 (100%)	7.3*e*-074	50	10
2.	Motif_2	7 (70%)	1.1*e*-046	50	7
3.	Motif_3	7 (70%)	2.0*e*-048	50	7
4.	Motif_4	9 (90%)	1.4*e*-046	50	9
5.	Motif_5	7 (70%)	7.2*e*-033	41	7

^a^Probability of finding an equally well-conserved motif in random sequences.

**Table 4:** List of matching candidates for EXPREG transcription factor (TF).

SN	Candidate of TF	Strains showed motif sequence binding	GC (%)	Regulatory roles				Statistical significance
				Activation (%)	Repression (%)	Dual (%)	Not specified (%)	
1.	*CRP*	*Y. pestis*	46.88	0	100	0	0	2.11*e*+00
2.	*PhhR_*	*P. putida*	46.67	90	10	0	0	2.29*e*+00
3.	*VqsM_*	*P. aeruginosa*	59.33	7	0	0	92	3.43*e*+00
4.	*CodY*	*B. anthracis*	20.41	0	0	0	100	3.99*e*+00
5.	*Fur*	*P. syringae*	40.25	0	13	0	85	4.88*e*+00
6.	*EspR*	*M. tuberculosis*	52.83	0	0	0	100	5.95*e*+00
7.	*MatP*	*E. coli*	47.23	0	0	0	100	6.75*e*+00
8.	*CcpA*	*C. difficile*	26.32	9	36	0	53	6.87*e*+00
9.	*GlxR*	*C. glutamicum*	46.55	0	100	0	0	7.38*e*+00
10.	*Lrp*	*E. coli*	40.00	1	1	0	97	7.91*e*+00
11.	*CtrA*	*C. crescentus*	28.95	0	0	20	80	9.29*e*+00

*CRP*: cAMP receptor protein; *PhhR*: phenylalanine hydroxylase regulator; *VqsM*: virulence and QS modulator; *Fur*: Ferric uptake regulation protein; *CcpA*: Catabolite control protein A; *MatP*: membrane-associated transfer protein; *LrP*: leucine-responsive regulatory protein.

**Table 5:** List of match candidates of EXPREG transcription Confirmation Factor (TCF).

SN	Candidate TF	Strains that show motif sequence binding	GC (%)	TF confirmation mode				Not specified (%)	Statistical significance
				Monomer (%)	Dimer (%)	Tetramer (%)	Other (%)		
1.	*CRP*	*Y. pestis*	46.88	0	0	0	0	100	2.11*e*+00
2.	*PhhR*	*P. putida*	46.67	0	100	0	0	0	2.29*e*+00
3.	*VqsM*	*P. aeruginosa*	59.33	0	0	0	0	100	3.43*e*+00
4.	*CodY*	*B. anthracis*	20.41	0	0	0	0	100	3.99*e*+00
5.	*Fur*	*P. syringae*	40.25	0	100	0	0	0	4.88*e*+00
6.	*EspR*	*M .tuberculosis*	52.83	0	100	0	0	0	5.95*e*+00
7.	*MatP*	*E. coli*	4723	0	100	0	0	0	6.75*e*+00
8.	*CcpA*	*C. difficile*	26.32	0	0	0	0	100	6.87*e*+00
9.	*GlxR*	*C. glutamicum*	46.55	0	0	0	0	100	7.38*e*+00
10.	*Lrp*	*E. coli*	40.00	0	96	0		3	7.91*e*+00
11.	*CtrA*	*C. crescentus*	28.95	0	0	0	0	100	9.29*e*+00

*CRP*: cAMP receptor protein; *PhhR*: phenylalanine hydroxylase regulator; *VqsM*: virulence and QS modulator; *Fur*: Ferric uptake regulation protein; *CcpA*: Catabolite control protein A; *MatP*: membrane-associated transfer protein; *LrP*: leucine-responsive regulatory protein.

**Table 6:** CpG islands identified at both promoter and gene body regions.

SN	Gene ID	Gene body regions					Promoter regions				
		Start	End	Length	No. of CpG found	GC%	Start	End	Length	No. of CpG found	GC%
1.	46432416	8	631	624	1	57	11	1970	1960	1	58
2.	66762507	1	631	631	1	50	1	1987	1987	1	62
3.	66762509	–	–	–	–	–	–	–	–	–	–
4.	69747978	–	–	–	–	–	1	1978	1978	1	54
5.	69747981	–	–	–	–	–	1	1990	1990	1	64
6.	69751968	–	–	–	–	–	–	–	–	–	–
7.	69751970	1	1639	1639	1	53	6	1997	1992	1	60
8.	69751971	–	–	–	–	–	1	1964	1965	1	57
9.	69751972	–	–	–	–	–	1	1996	1996	1	70
10.	69751974	–	–	–	–	–	–	–	–	–	–

Database of CpG islands and analytical tools [16].

**Table 7:** *MspI* cutting sites and fragment sizes in promoter regions.

Region	Corresponding sequences	Nucleotide positions of *MSPI* sites	Fragment size between 40 and 220 bp
Promoter region	Prom_69751970	12 (102, 121, 485, 528, 935, 941, 969, 991, 1324, 1346, 1565, 1877)	43
	Prom_66762507	15 (238, 382, 392, 412, 529, 661, 1021, 1027, 1172, 1230, 1384, 1504, 1579, 1614, 1818)	144, 117, 132, 145, 58, 154, 120, 75, 204
	Prom_66762509	11 (295, 497, 1059, 1165, 1221, 1542, 1558, 1577, 1758, 1797, 1960)	202, 106, 56, 181, 163
	Prom_69747981	19 (35, 317, 433, 445, 470, 589, 768, 901, 1027, 1045, 1167, 1225, 1379, 1499, 1547, 1574, 1609, 1813, 1976)	116, 119, 179, 133, 126, 122, 58, 154, 120, 48, 204, 163
	Prom_69751968	23 (52, 173, 226, 407, 655, 666, 684, 806, 854, 864, 976, 1018, 1138, 1186, 1213, 1248, 1452, 1659, 1676, 1729, 1744, 1848, 1975)	121, 53, 181, 122, 48, 112, 42, 120, 48, 207, 53, 104, 127
	Prom_69751971	14 (6, 160, 185, 344, 363, 727, 770, 1177, 1183, 1211, 1233, 1566, 1588, 1807)	154, 159, 219
	Prom_69751974	15 (40, 152, 162, 210, 332, 350, 361, 609, 790, 843, 964, 1164, 1476, 1695, 1717)	112, 48, 122, 181, 53, 121, 200, 212, 219
	Prom_69751972	15 (63, 271, 284, 438, 463, 622, 641, 1005, 1048, 1455, 1461, 1489, 1511, 1844, 1866)	208, 154, 159, 43
	Prom_69747978	6 (104, 306, 666, 700, 715, 1877)	104
	Prom_46432416	12 (129, 347, 562, 1179, 1361, 1536, 1556, 1591, 1763, 1795, 1836, 1958)	218, 215, 182, 175, 172, 41, 122

**Table 8:** *MspI* cutting sites and fragment sizes in gene body regions.

Region	Corresponding sequences	Nucleotide positions of *MSPI* sites	Fragment size between 40 and 220 bp
Gene bodies	ORF 69751970	17 (77, 198, 251, 432, 680, 691, 709, 831, 879, 889, 1001, 1043, 1163, 1211, 1238, 1273, 1477)	121, 53, 181, 122, 48, 112, 42, 120, 48, 204
	ORF 66762507	3 (54, 279, 319)	40
	ORF 66762509	1 (403)	−
	ORF 69747981	5 (21, 38, 91, 210, 337)	53, 119, 127
	ORF 69751968	4 (47, 119, 131, 179)	72, 48
	ORF 69751971	1 (119)	−
	ORF 69751974	4 (50,72 100, 106)	−
	ORF 69751972	1 (85)	−
	ORF 69747978	3 (17, 210, 232)	193
	ORF 46432416	1 (636)	−

## Data Availability

The data was extracted from NCBI and can be obtained from the corresponding author.

## Abbreviations

TSS: Transcriptional start site
NNPP: Neural Network Promoter Prediction
TFs: Transcriptional factors
CpG: Cytosine phosphate guanine
NCBI: National Center for Biotechnology Information.
